# NaF-PET/CT imaging of atherosclerosis in type 2 diabetes: Associations with sex and history of cardiovascular events in a 2-year follow-up study

**DOI:** 10.1007/s00259-025-07353-5

**Published:** 2025-06-05

**Authors:** Reza Piri, Jacob Volmer Stidsen, Jens Steen Nielsen, Jan Erik Henriksen, Reimar Wernich Thomsen, Thomas Bastholm Olesen, Manan Pareek, Axel Cosmus Pyndt Diederichsen, Michael Hecht Olsen, Poul Flemming Høilund-Carlsen

**Affiliations:** 1 Department of Nuclear Medicine, Odense University Hospital, Odense, Denmark; 2 Department of Clinical Research, University of Southern Denmark, Odense, Denmark; 3 Department of Radiology, Rigshospital, Copenhagen, Denmark; 4 Department of Endocrinology, Steno Diabetes Centre Odense, Odense University Hospital, Odense, Denmark; 5 Department of Clinical Epidemiology, Aarhus University and Aarhus University Hospital, Aarhus, Denmark; 6 Department of Cardiology, Copenhagen University Hospital—Herlev and Gentofte, Copenhagen, Denmark; 7 Center for Translational Cardiology and Pragmatic Randomized Trials, Department of Biomedical Sciences, Faculty of Health and Medical Sciences, University of Copenhagen, Copenhagen, Denmark; 8 Department of Cardiology, Odense University Hospital, Odense, Denmark; 9 Department of Medicine 1 and Steno Diabetes Center Zealand, Holbaek Hospital, Holbaek, Denmark; 10 Department of Clinical Medicine, University of Copenhagen, Copenhagen, Denmark

**Keywords:** Positron emission tomography, Microcalcification, Cardiovascular disease, Atherosclerosis, Diabetes, Sex

## Abstract

**Purpose:**

This study aims to evaluate the progression of atherosclerosis over two years in individuals with type 2 diabetes mellitus (T2DM) using sodium fluoride (NaF) positron emission tomography/computed tomography (PET/CT) and to assess its relationship with sex and history of cardiovascular diseases (CVD).

**Methods:**

A total of 112 T2DM patients from the project “Specialist supervised individualized multifactorial treatment of new clinically diagnosed type 2 diabetes in general practice” (IDA) were included, divided into four groups based on sex and history of CVD events. All underwent NaF-PET/CT at baseline and after two years. Standardized uptake values (SUVs) were retrieved for the carotids, the heart, and the aorta. Partial correlations between NaF uptake and age, body mass index (BMI), low-density lipoprotein (LDL) cholesterol, blood pressure, and CT-derived coronary calcium score were evaluated, adjusted for history of CVD events and sex.

**Results:**

Females without CVD exhibited significantly higher NaF uptake than males without CVD, particularly in the aorta, SUVmean 1.62 ± 0.20 vs. 1.41 ± 0.20, *p* < 0.001. Positive correlations were found between NaF uptake in the aorta and age (*r* = 0.29, *p* = 0.002), BMI (*r* = 0.33, *p* < 0.001), and weight (*r* = 0.28, *p* = 0.002), but not LDL, blood pressure, or coronary artery calcium scores. Longitudinal analysis showed a significant average increase in NaF uptake of 0.07 (*p* = 0.04) in aorta over time in females without CVD, with no significant changes in other groups.

**Conclusion:**

Our study suggests that microcalcification, indicated by NaF uptake, progresses differently between sexes. Menopausal females, particularly those without CVD, show higher uptake compared to others.

**Supplementary Information:**

The online version contains supplementary material available at 10.1007/s00259-025-07353-5.

## Introduction

Despite advancements in medical science, atherosclerosis-related cardiovascular and cerebrovascular diseases remain leading causes of death worldwide [1–4]. Notably, type 2 diabetes (T2DM) and male sex are significant risk factor for cardiovascular disease (CVD), substantially increasing the likelihood of cardiovascular events [5, 6]. Atherosclerosis, involving fatty deposits, cholesterol, and calcium in arterial walls, leads to plaque build-up that can obstruct blood flow and often goes undetected until advanced stages, highlighting the need for early diagnostic tools [7, 8]. The process begins with inflammation, attracting immune cells that form plaques, which calcify over time and become visible on imaging like computed tomography (CT) and magnetic resonance imaging (MRI). However, these methods typically detect only advanced stages of atherosclerosis [7]. Positron emission tomography (PET), especially with the radiotracer ^l8^F-sodium fluoride (NaF), allows for earlier detection of atherosclerosis by identifying microcalcification, an early marker of plaque formation [9–13].

**Fig. 1:**
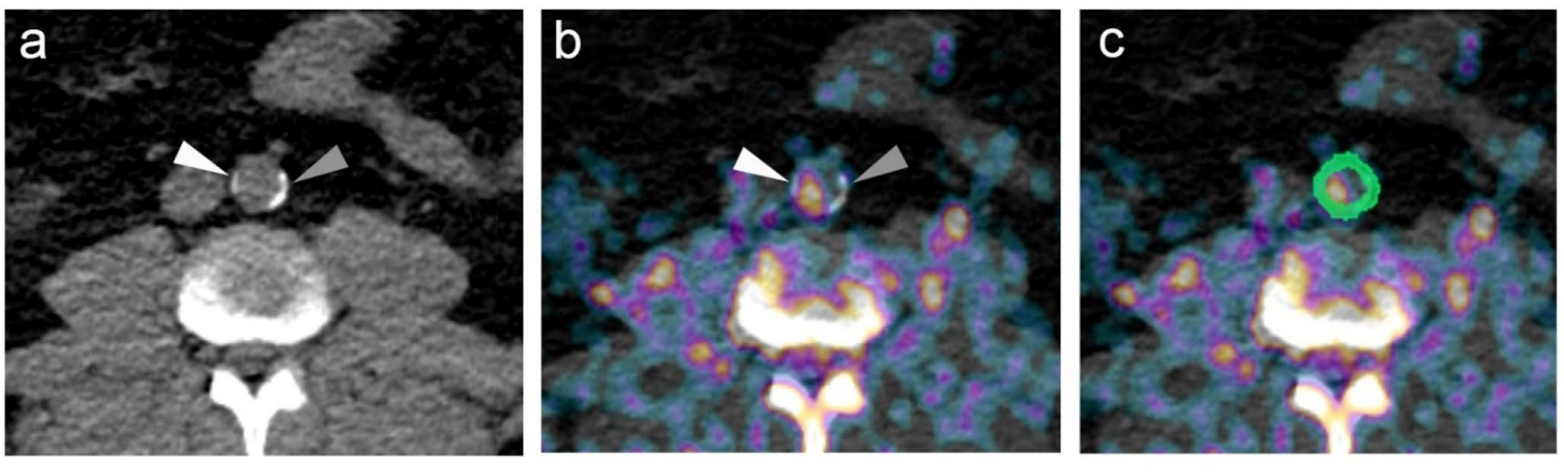
**a** Transversal CT scan of the abdominal aorta, where the white arrowhead indicates a smaller calcification, while the gray arrowhead points to a larger, denser calcification. **b** The same image overlaid with NaF-PET shows high NaF uptake in an area between the two CT-detectable calcifications, partially overlaying the smaller, less dense calcification (white arrowhead), while the larger, denser calcification (grey arrowhead) shows only a small amount of NaF uptake in the center where there is the least calcification on CT. **c** Segmentation of arterial wall performed by artificial intelligence shown in green color

In atherosclerotic plaques, local necrosis can lead to the development of vacuoles and the deposition of hydroxyapatite crystals – microcalcification – within smooth muscle cells, marking the early stages of mineralization in the arterial wall [14, 15]. NaF has a high affinity for hydroxyapatite and binds selectively to these crystals [14, 16]. This property makes NaF-PET imaging particularly useful for detecting early microcalcification, as it highlights areas where hydroxyapatite deposits are present, offering valuable insights into the progression of atherosclerosis. Microcalcification (size < 50 μm) is distinct from macrocalcification (size > 200 μm), which occurs in more advanced atherosclerotic plaques [14]. As shown in Fig. 1, the smaller, less dense calcification exhibits higher NaF uptake, whereas the larger, denser calcification demonstrates lower uptake. Macrocalcification is typically assessed using metrics such as the coronary artery calcium (CAC) score derived from CT scans, and its presence signifies a later stage of plaque development following microcalcification [17, 18].

This study aimed to evaluate atherosclerosis with NaF-PET/CT in individuals with T2DM over two years, and to assess its relationship with sex and history of cardiovascular event. By assessing changes in NaF uptake in the carotid arteries, heart, and aorta over two years, we aimed to better understand the progression of atherosclerosis and its relationship to cardiovascular risk factors in this category of patients.

## Methods

### Study population

This study is a sub-study of “Specialist supervised Individualized multifactorial treatment of new clinically diagnosed type 2 diabetes in general practice” (IDA) project, focusing on individuals with T2DM [19]. This project investigates an individualized treatment approach for T2DM as an alternative to the conventional “treat-to-target” strategy, which often leads to polypharmacy and low patient adherence. The inclusion criteria for the study were as follows: Participants were required to be members of the Danish Centre for Strategic Research in Type 2 Diabetes (DD2) cohort and registered with a general practitioner participating in the IDA study. The DD2 cohort consists of patients newly diagnosed with Type 2 Diabetes from across Denmark. The enrollment process has been described in detail elsewhere [20]: briefly, clinical providers identify eligible patients during routine clinical practice, inform them about the DD2 project, and obtain written informed consent from those interested in participating. Eligible participants were further required to have a life expectancy exceeding two years, not be enrolled in other clinical trials, and be willing to provide written informed consent. The primary objective is to assess its impact on micro- and macrovascular complications, hypoglycemia, cancer, and overall mortality over a 10-year period. The project also aim to improve quality of life, reduce medication use, and enhance cost-effectiveness in diabetes care. A total of 112 participants underwent NaF-PET/CT scans at baseline and 101 participants again after two years. The selection criteria included a clinical diagnosis of T2DM.

## Imaging procedure and analysis

The NaF-PET/CT scans were conducted to evaluate the burden of atherosclerosis in the cardiovascular system. Imaging followed a formerly established protocol, including the administration of approximately 2.2 MBq/kg of 18 F-NaF [21]. Scans were performed 90 min post-injection using a hybrid PET/CT system [21]. For the automated analysis of the images, we used the RECOMIA platform, which employs artificial intelligence (AI) and convolutional neural networks (CNNs) for segmentation [22–25]. The CNN-based segmentation was performed using a model similar to 3D U-Net, designed to process the input image on multiple resolutions to extract detailed features. This model includes convolutional and rectified linear unit layers, max-pooling downsampling, upconvolution upsampling, and skip connections. An example of segmentation of arterial wall is shown in Fig. 1c. Standardized uptake values (SUVs) were calculated to quantify NaF uptake in the arterial walls. Volume of interest (VOIs) were defined for the carotid arteries, heart and segments of the aorta (arch, thoracic, and abdominal). SUVmean (mean standardized uptake value) refers to the average tracer uptake within a VOI, while SUVtotal represents the overall uptake in the VOI, i.e., the product of SUVmean and the volume of the region. The analysis aimed to capture the full extent of the arterial wall, avoiding adjacent structures such as vertebral bones that could interfere with measurements [26] due to the very high affinity of NaF to hydroxyapatite [14].

### Coronary artery calcium score calculation

Non-contrast ECG-gated CT scans were used to assess CAC scores. Image reconstruction was performed with a 0.5 mm slice thickness, and CAC scoring was analyzed using the Agatston method with Syngo.CT CaScoring software (Siemens Healthcare). Voxels with an attenuation of ≥ 130 HU were segmented, and lesion-specific scores were calculated based on lesion area and a density-weighting factor. The total CAC score reflects the extent of coronary calcification and serves as an indicator of cardiovascular risk.

### Data collection

Demographic data, including age, sex, and medical history, were collected at baseline. Systolic and diastolic blood pressure were obtained by unobserved automatic measurement every 3 min for 30 min in the sitting position. Height and weight were obtained by standard procedures and BMI calculated. Low-density lipoprotein cholesterol (LDL) levels, hemoglobin A1c (HbA1c) and urine albumin-to-creatinine ratio (UACR) were obtained from routine clinical measurements closest to the visit, and CAC scores were recorded. History of CVD was obtained by clinical interview and journal audit at baseline and at 2-year follow-up. CVD was defined as cerebrovascular infarction, myocardial infarction, coronary artery bypass graft, percutaneous coronary intervention, extracardial revascularization procedures, confirmed arterial narrowing of > 50% or ankle-arm-index < 0.9. The use of medications was recorded at baseline and follow-up. For each patient, information on the use of cardiovascular, antihypertensive, antidiabetic, and lipid-lowering medications was collected. Specifically, the medication groups included statins, angiotensin-converting enzyme inhibitors, angiotensin II receptor blockers, aldosterone receptor antagonists, beta blockers, calcium channel blockers, dipeptidyl peptidase-4 inhibitors, glucagon-like peptide-1 receptor agonists, proprotein convertase subtilisin/kexin type 9 inhibitors, sodium-glucose co-transporter-2 inhibitors, sulfonylureas, acarbose, pioglitazone, basal insulin, meal insulin, mixed insulin, loop diuretics, thiazide diuretics, renin inhibitors, fibrates, anion exchangers, and ezetimibe. Medication use meant that the patient was actively taking the medication at the time of assessment. Changes in medication use between baseline and follow-up were categorized as initiation (no use at baseline but use at follow-up) or discontinuation (use at baseline but no use at follow-up).

### Statistical analysis

Descriptive statistics were calculated for four groups based on baseline history of CVD and sex. For variables with a normal distribution, the means and standard deviations (SD) were reported. For non-normal distributions, the medians and interquartile ranges (IQR) were used. Normality was tested using the Shapiro-Wilk test. To assess whether the use of cardiovascular, antidiabetic, and lipid-lowering medications influenced NaF uptake in the aorta, multiple linear regression models were applied. Separate models were constructed for baseline and follow-up NaF uptake, with medication use included as independent variable. To compare groups, one-way analysis of variance (ANOVA) was applied for normally distributed variables, with Tukey’s test used for post hoc comparisons. For non-normally distributed variables, the Kruskal-Wallis test was performed, followed by pairwise Mann-Whitney U tests when significant differences were found. Significant group differences were highlighted. Partial correlations adjusted for sex and CVD status at baseline were calculated to explore relationships between SUV variables and demographic or clinical data. Change in SUV variables from baseline to 2-year follow-up were also explored by partial correlation adjusted for sex and history of CVD at baseline. Correlation coefficients (r) and their p-values were reported in the format: r (p-value). A *p*-value < 0.05 was considered statistically significant. Analyses were performed using SPSS version 19.0 (SPSS Inc., Chicago, IL, USA). Python (Spyder IDE) with pandas was used to generate the plots.

## Results

Demographics and paraclinical findings for all the participants, showed a mean participant age of 63.3 ± 10.2 years, with 40 (35.7%) female participants; Table 1 shows demographics and paraclinical findings categorized by CVD history and sex at baseline. A comparison of SUVmean and SUVtotal values across cardiovascular sections in the four patient groups at baseline revealed significant differences in SUVmean across all segments, with females generally showing higher values than males (Table 2). In patients without a history of CVD, the SUVmean in the aorta was significantly higher in women compared with men (1.62 ± 0.20 vs. 1.41 ± 0.20, *p* < 0.001). However, this difference was reverse for SUVtotal and in most cases statistically significant; in patients without a history of CVD, the SUVtotal in the heart was significantly higher in men compared with women (820.12 ± 217.41 vs. 708.46 ± 175.14, *p* = 0.02). Differences between groups with and without CVD history was not significant in most of the cases. Additionally, smoking status and duration of T2DM did not affect NaF uptake in cardiovascular segments. Supplementary Material 1 provides additional details on medication use and changes observed between baseline and follow-up. In the multiple regression analyses, the use of cardiovascular, antidiabetic, and lipid-lowering medications showed no significant association with NaF uptake in the aorta at baseline or follow-up.

**Table 1:**
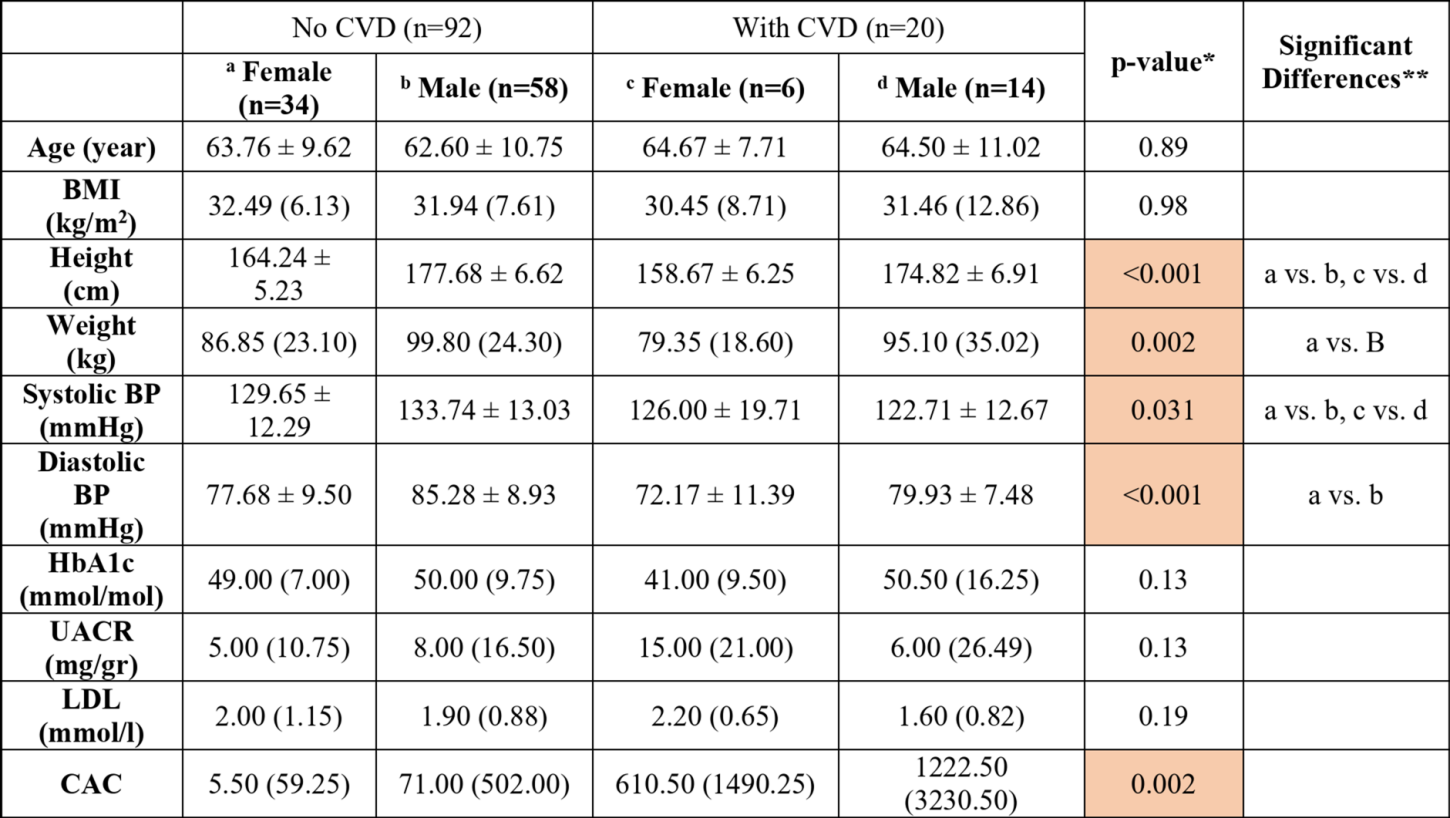
Baseline demographics and paraclinical characteristics by history of cardiovascular disease (CVD) and sex.*

*ANOVA or Kruskal Wallis test

**Groups are marked by superscripted alphabets. In case of significant post-hoc difference, the different groups are indicated. The differences with clinical importance were a vs. b, a vs. c, b vs. d and c vs. d

*BMI* body mass index, *BP* blood pressure, *HbA1c* glycated hemoglobin, *UACR* urinary albumin-to-creatinine ratio, *LDL* low-density lipoprotein, *CAC* coronary artery calcium

*Significant positive correlations are highlighted in orange,

**Table 2:**
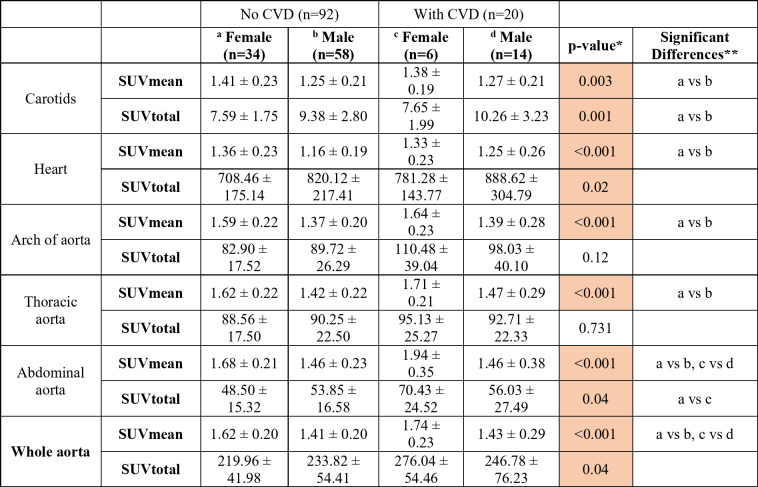
Comparison of SUVmean and SUVtotal across cardiovascular segments between patient groups at baseline.*

*ANOVA or Kruskal Wallis test

**Groups are marked by superscripted alphabets. In case of significant post-hoc difference, the different groups are indicated. The differences with clinical importance were a vs. b, a vs. c, b vs. d and c vs. d

*BMI* body mass index, *BP* blood pressure, *HbA1c* glycated hemoglobin, *UACR* urinary albumin-to-creatinine ratio, *LDL* low-density lipoprotein, *CAC* coronary artery calcium

*Significant positive correlations are highlighted in orange,

Several significant relationships between NaF uptake and patient characteristics were identified in the partial correlation analysis, adjusted for CVD history and sex at baseline (Table 3); age, BMI and weight correlated positively with most of the NaF uptake variables; there was a positive correlation between SUVmean in the whole aorta with age (*r* = 0.29, *p* = 0.002) and BMI (*r* = 0.33, *p* < 0.001). However, NaF uptake was negatively correlated with diastolic blood pressure, LDL, and HbA1c levels. Table 4 shows the partial correlation analysis adjusted for CVD history and sex between changes in NaF uptake (baseline and follow-up) and patient characteristics.

**Table 3:**
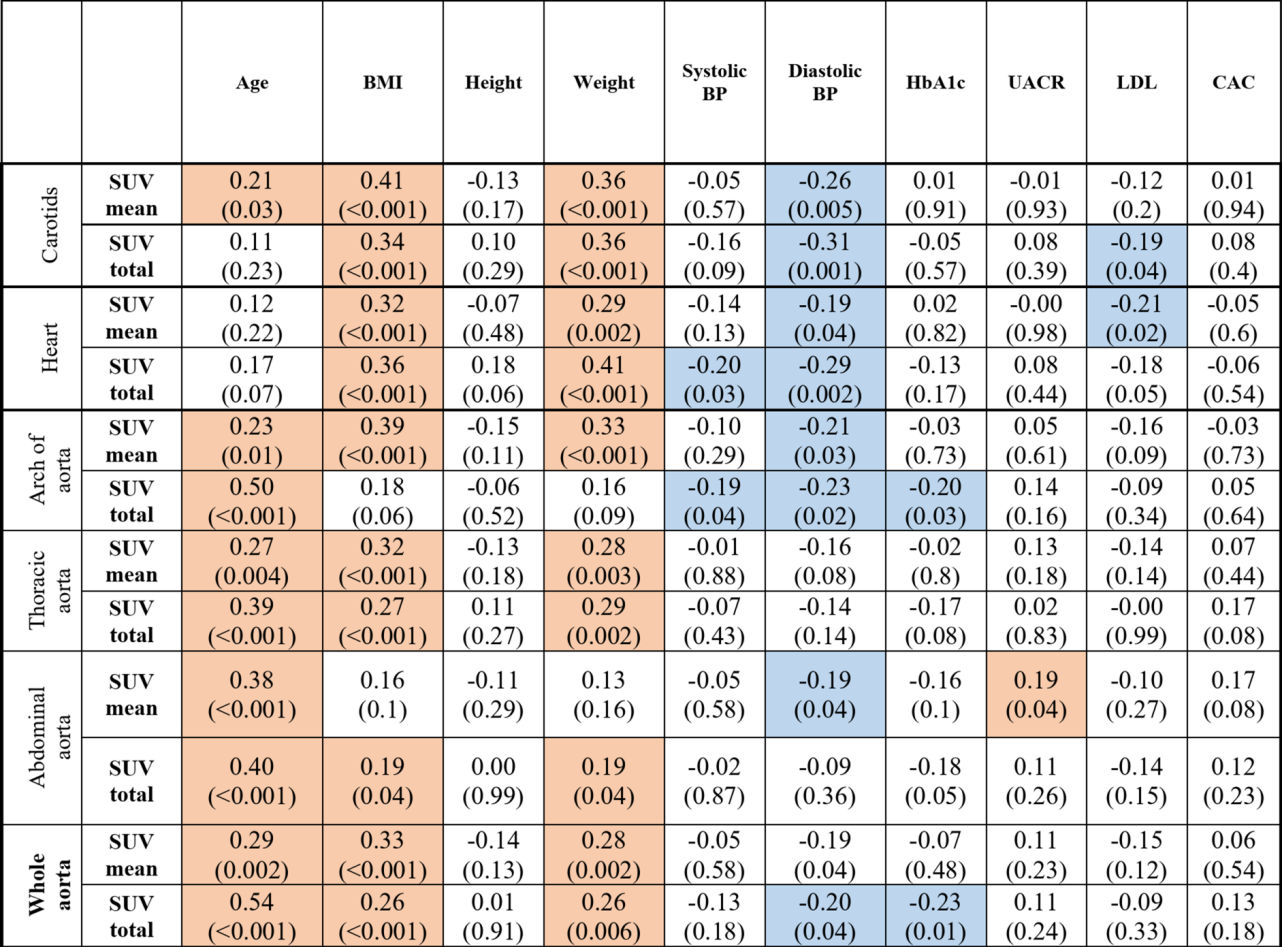
Partial correlations between NaF uptake (SUVmean and SUVtotal) and patient characteristics adjusted for CVD and sex in at baseline.*

*BMI* body mass index, *BP* blood pressure, *HbA1c* glycated hemoglobin, *UACR* urinary albumin-to-creatinine ratio, *LDL* low-density lipoprotein, *CAC* coronary artery calcium

*Significant positive correlations are highlighted in orange, and significant negative correlations are highlighted in blue

**Table 4:**
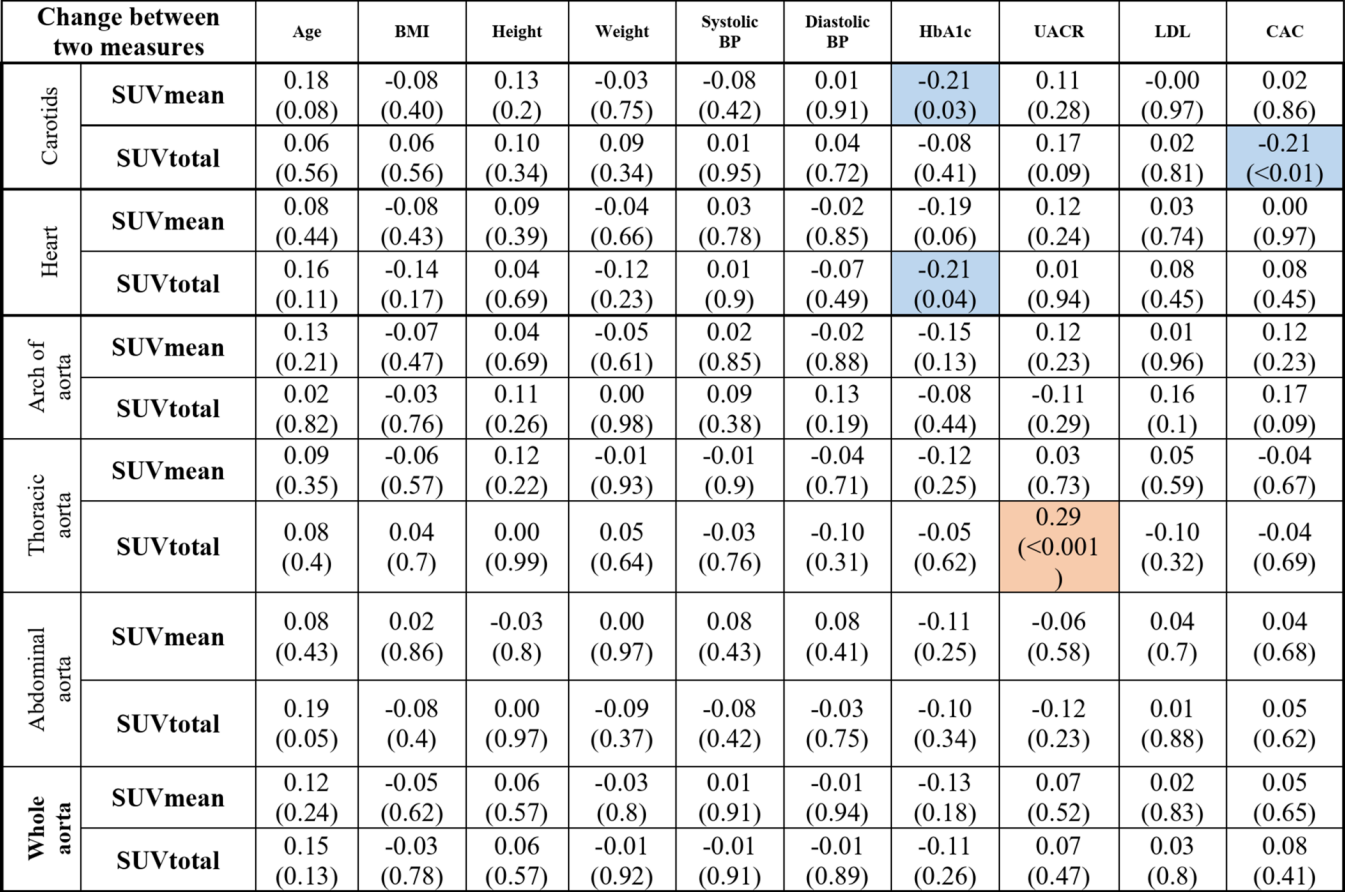
Partial correlations between NaF uptake change from baseline to 2 year follow-up (SUVmean and SUVtotal) and patient characteristics adjusted for CVD and sex.*

*BMI* body mass index, *BP* blood pressure, *HbA1c* glycated hemoglobin, *UACR* urinary albumin-to-creatinine ratio, *LDL* low-density lipoprotein, *CAC* coronary artery calcium

*Significant positive correlations are highlighted in orange, and significant negative correlations are highlighted in blue

**Fig. 2:**
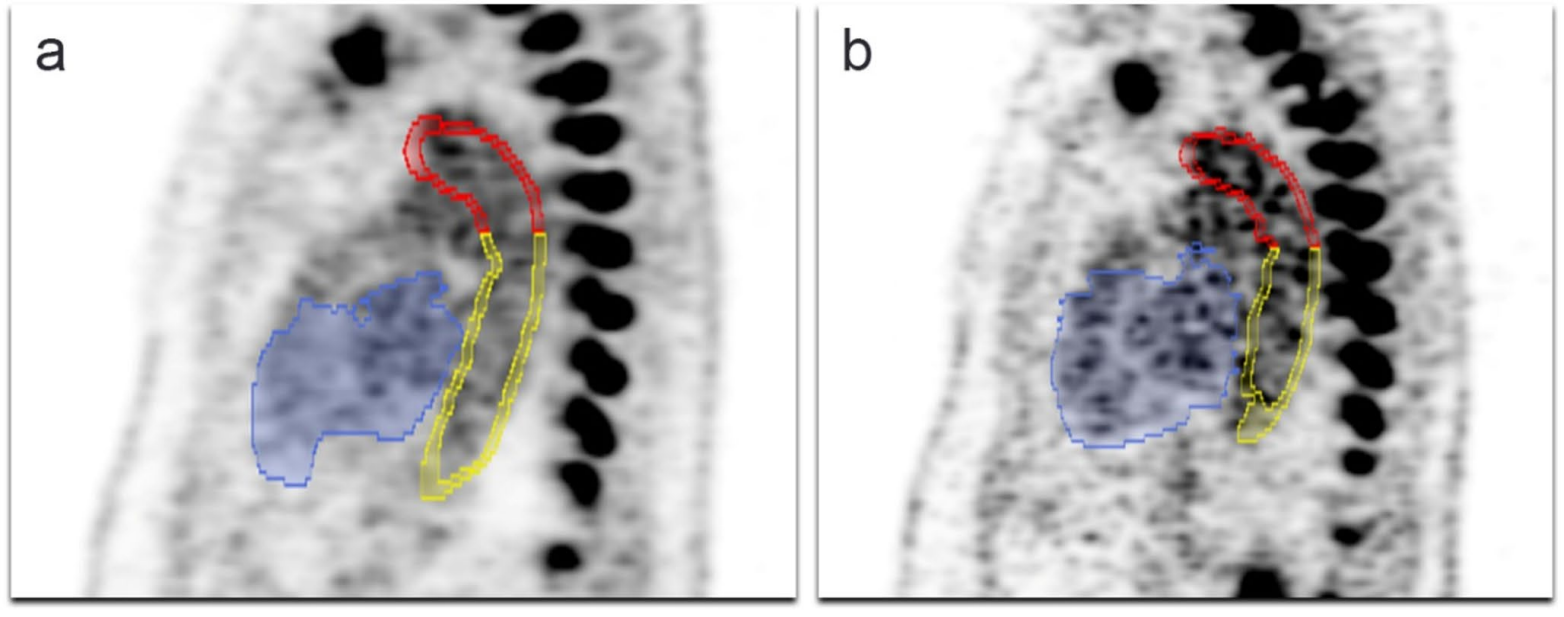
Sagittal NaF-PET images of the thorax from a patient at (**a**) baseline and (**b**) follow-up showing on both occasions approximately the same sagittal slice through the cardiovascular system, including part of the heart (segmented in blue), the aortic arch (segmented in red), and the thoracic aorta (segmented in yellow). NaF uptake is displayed in grayscale, with darker pixels indicating higher uptake. Even if not readily apparent by visual inspection, the difference is clearly there according to sensitive AI-based quantification

The mean change in SUVmean and SUVtotal values between baseline and follow-up across the four patient groups—females and males with and without CVD history —is outlined in Table 5. However, the mean of the changes were positive, and it was only the females with no history of CVD who had significantly higher NaF uptake values at follow-up compared with baseline, except in the carotids; the mean changes from baseline to follow-up demonstrated a significant increase in both SUVmean (0.07 ± 0.17, *p* = 0.04) and SUVtotal (13.4 ± 22.96, *p* = < 0.001) in the aorta after two years. An example of increased NaF uptake in the cardiovascular system from baseline to follow-up is shown in Fig. 2.

**Table 5:**
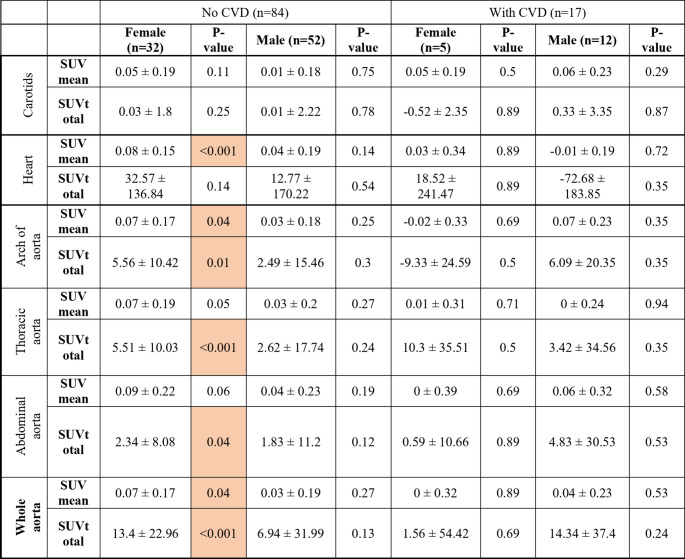
Mean changes from baseline to follow-up in SUVmean and SUVtotal across cardiovascular segments stratified by CVD and sex.*

*Significant positive correlations are highlighted in orange

## Discussion

Our findings underscore that microcalcification, as measured by NaF uptake, is most pronounced in postmenopausal women. This observation aligns with the hypothesis that the loss of estrogen’s protective effects after menopause accelerates the atherosclerotic process, necessitating these women to ‘catch up’ on the vascular changes that estrogen had previously delayed [27]. This may explain the significant NaF uptake seen in females without overt CVD history in our cohort. This sex-based difference was significant for SUVmean, although not present for SUVtotal, as larger cardiovascular volumes in males may offset the total uptake differences.

Interestingly, NaF uptake appeared to be independent of existing CVD and showed positive correlations with age and BMI, both of which are established cardiovascular risk factors that are inherently challenging to manage or reverse [28]. Conversely, NaF uptake demonstrated a negative relationship with diastolic blood pressure, HbA1c, and LDL levels, which are strongly influenced by medical treatment in T2DM patients. These findings suggest that therapeutic interventions targeting glycemic control and lipid levels may alter traditional positive associations between these factors and atherosclerosis markers. This unexpected finding may also partly be explained by the relatively low variability in these parameters within the cohort, as well as by the intensive clinical management typical for high-risk diabetic patients, including the widespread use of lipid-lowering and antihypertensive therapies. These factors could have attenuated the expected relationships between risk markers and NaF uptake. 2-year change in SUVmean showed an overall increase in NaF uptake from baseline to follow-up, with significant changes observed only in females without CVD.

Our finding of higher NaF uptake in females, particularly those without CVD, is noteworthy. It contrasts with the conventional understanding that male sex is associated with a higher risk of atherosclerosis and cardiovascular diseases [29, 30]. Studies such as those by Detrano et al. and Shaw et al. have shown that men tend to have higher CAC scores, reflecting a greater burden of macrocalcification and a higher incidence of coronary events [18]. This suggests that microcalcification, as identified by NaF uptake, may manifest differently between sexes, with females of similar age as men possibly having earlier stages of atherosclerosis.

The observed difference may align with the findings that women, compared with men, often have fewer plaque ruptures, shorter lesion lengths, and smaller necrotic cores when presenting with acute coronary syndrome [29]. Moreover, women tend to exhibit more diffuse atherosclerosis, while men often develop more focal, obstructive coronary artery disease (CAD) [31]. Pepine et al. have shown that women frequently present with ischemia despite non-obstructive CAD, suggesting a strong role for microvascular dysfunction in female patients [32]. Furthermore, women tend to have more extensive, numerous, or larger atherosclerotic coronary lesions that, however, are macroscopically less calcified [18]. Women are also more prone to plaque erosion, while men are more likely to experience plaque rupture [18, 31]. This may explain the higher NaF uptake in females without CVD, as microcalcifications often occur in the presence of diffuse atherosclerosis and microvascular disease.

Intravascular ultrasound studies support these findings, demonstrating that women, even in the absence of obstructive CAD, exhibit more widespread microcalcification. A study by Khuddus et al. indicated that up to 80% of women with stable chest pain, but without obstructive CAD, had detectable atherosclerosis via intravascular ultrasound, underscoring the importance of microcalcifications in this population [33]. Our observation of higher NaF uptake in females without CVD aligns with this understanding, suggesting that microcalcification plays a crucial role in early atherosclerosis in women.

Additionally, computational analyses using coronary CT angiography have shed light on sex-specific differences in coronary anatomy and physiology [34]. Women typically have smaller coronary arteries, which may exacerbate the effects of plaque burden. The ratio of total epicardial coronary arterial lumen volume to left ventricular mass has been associated with microvascular disease. This provides a possible explanation for the higher NaF uptake in women, as they may be more susceptible to plaque load even with a lower overall burden compared to men. It has also been demonstrated that women were less likely to have obstructive CAD but more likely to experience ischemia due to microvascular disease, further explaining the NaF uptake in women without CVD [35].

In comparing baseline and follow-up NaF uptake, we observed a significant increase only in females without CVD. This may be linked to the fact that many, if not all, of the women in our study were post-menopausal, a stage associated with accelerated atherosclerosis progression. Research has shown that calcification tends to develop about a decade later in women compared with men, with the gap narrowing after menopause due to the loss of estrogen’s protective effects [36]. Thus, as mentioned, the observed increase in NaF uptake in post-menopausal women could reflect the accelerated progression of microcalcification when the protective effect of estrogen fades away after menopause.

T2DM may also play a role in the higher NaF uptake observed in women without CVD history. T2DM is recognized as a more significant risk factor for CAD in women than in men. Thus, Peters et al. found that T2DM increases the risk of coronary events in women to a greater extent than in men, potentially explaining the significant NaF uptake in diabetic women without CVD [37]. The lack of a similar statistically significant trend in women with CVD may be due to the smaller sample size or the presence of more advanced macrocalcifications, which could overshadow microcalcification in this group.

## Limitations

Key limitations of our study include a small sample size of CVD patients, which may have reduced the power to detect other significant differences between groups, and the exclusive inclusion of T2DM patients, limiting generalizability to populations without diabetes. The absence of a non-diabetic control group further restricted our ability to assess whether NaF uptake patterns are specific to diabetes. Additionally, using SUVmean and SUVtotal in large cardiovascular segments may have limited sensitivity for detecting localized NaF uptake.

## Conclusion

This study identifies significant sex differences in NaF uptake, with higher microcalcification levels observed in T2DM females without CVD compared to males. While macrocalcification, typically assessed by CAC scores, is more prominent in men, microcalcification appears more pronounced in menopausal women with T2DM. NaF uptake was associated with age and BMI and negatively correlated with diastolic blood pressure, HbA1c, and LDL. Future studies with larger, more diverse cohorts and a focus on localized microcalcification using NaF PET/CT may enhance understanding of early atherosclerosis and its clinical implications.

## Electronic supplementary material

Below is the link to the electronic supplementary material.

Supplementary Material 1 (438 KB)

## Data Availability

The datasets generated during and/or analysed during the current study are available from the corresponding author on reasonable request.
