# Supplementary material for: NaF-PET/CT imaging of atherosclerosis in type 2 diabetes: Associations with sex and history of cardiovascular events in a 2-year follow-up study
Source: Eur J Nucl Med Mol Imaging. 2025 Jun 5;52(13):5006–16. doi: 10.1007/s00259-025-07353-5 (PMC12589346; doi:10.1007/s00259-025-07353-5)
Supplement: Supplementary file 1 — Supplementary Material 1 (438 KB) [file 259_2025_7353_MOESM1_ESM.docx]

**NaF-PET/CT Imaging of Atherosclerosis in Type 2 Diabetes: Associations with Sex and History of Cardiovascular Events in a 2-Year Follow-up Study**

**Journal name:** European Journal of Nuclear Medicine and Molecular Imaging

Reza Piri^1,2,3^, Jacob Volmer Stidsen^4^, Jens Steen Nielsen^4^, Jan Erik Henriksen^4^, Reimar Wernich Thomsen^5^, Thomas Bastholm Olesen^4^, Manan Pareek^6,7^, Axel Cosmus Pyndt Diederichsen^8^, Michael Hecht Olsen^9,10^, and Poul Flemming Høilund-Carlsen^1,2^.

^1^ Department of Nuclear Medicine, Odense University Hospital, Odense, Denmark

^2^ Department of Clinical Research, University of Southern Denmark, Odense, Denmark

^3^ Department of Radiology, Rigshospital, Copenhagen, Denmark

^4^ Department of Endocrinology, Steno Diabetes Centre Odense, Odense University Hospital, Odense, Denmark

^5^ Department of Clinical Epidemiology, Aarhus University and Aarhus University Hospital, Aarhus, Denmark

^6^ Department of Cardiology, Copenhagen University Hospital—Herlev and Gentofte, Copenhagen, Denmark

^7^ Center for Translational Cardiology and Pragmatic Randomized Trials, Department of Biomedical Sciences, Faculty of Health and Medical Sciences, University of Copenhagen, Copenhagen, Denmark

^8^ Department of Cardiology, Odense University Hospital, Odense, Denmark

^9^ Department of Medicine 1 and Steno Diabetes Center Zealand, Holbæk Hospital, Holbæk, Denmark

^10^ Department of Clinical Medicine, University of Copenhagen, Copenhagen, Denmark

**Corresponding author:** Poul Flemming Høilund-Carlsen, Department of Nuclear Medicine, Odense University Hospital, Department of Clinical Research, University of Southern Denmark, Odense, Denmark. Email: pfhc@rsyd.dk


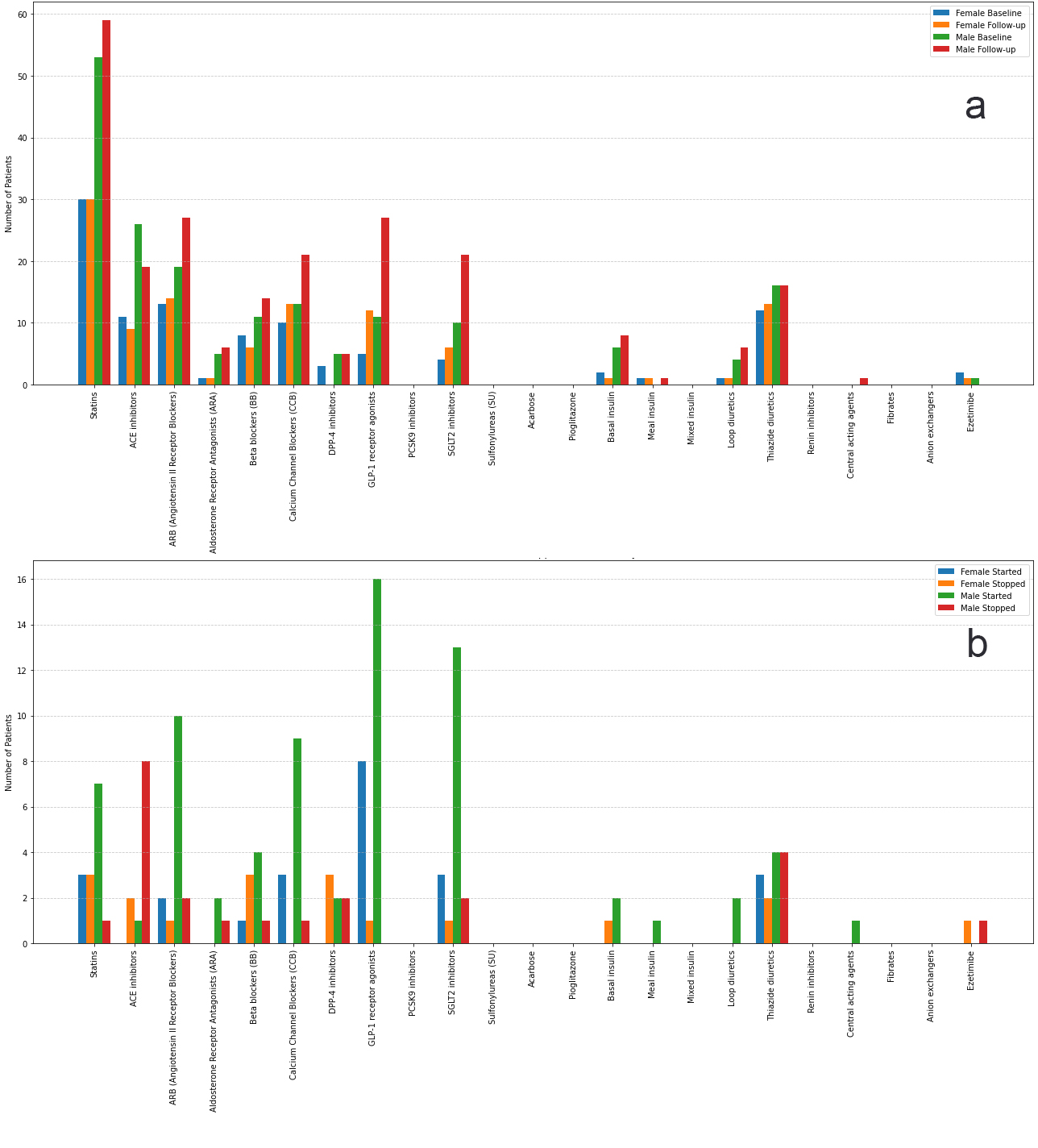
**Supplementary material 1.** Figure (a) shows the percentage of patients taking each medication at baseline and follow-up, separated by sex. For each medication, baseline and follow-up percentages are displayed side by side within each sex group. Figure (b) presents the number of patients who started or stopped each medication between baseline and follow-up, also categorized by sex. Starting medication is defined as not using the medication at baseline but using it at follow-up, while stopping medication is defined as using the medication at baseline but not using it at follow-up. **ACE** angiotensin-converting enzyme inhibitors, **ARB** angiotensin II receptor blockers, **ARA** aldosterone receptor antagonists, **BB** beta blockers, **CCB** calcium channel blockers, **DPP-4** **inhibitors** dipeptidyl peptidase-4 inhibitors, **GLP-1 receptor agonists** glucagon-like peptide-1 receptor agonists, **PCSK9 inhibitors** proprotein convertase subtilisin/kexin type 9 inhibitors, **SGLT2** inhibitors for sodium-glucose co-transporter-2 inhibitors, and **SU** sulfonylureas.
